# A Methodical Review on the Applications and Potentialities of Using Nanobiosensors for Disease Diagnosis

**DOI:** 10.1155/2022/1682502

**Published:** 2022-01-29

**Authors:** Kingsley Eghonghon Ukhurebor, Robert Birundu Onyancha, Uyiosa Osagie Aigbe, Gladys UK-Eghonghon, Rout George Kerry, Heri Septya Kusuma, Handoko Darmokoesoemo, Otolorin Adelaja Osibote, Vincent Aizebeoje Balogun

**Affiliations:** ^1^Department of Physics, Faculty of Science, Edo State University Uzairue, P.M.B. 04, Auchi, 312101 Edo State, Nigeria; ^2^Department of Physics and Space Science, School of Physical Sciences and Technology, Technical University of Kenya, P.O. Box 52428, 00200 Nairobi, Kenya; ^3^Department of Mathematics and Physics, Faculty of Applied Sciences, Cape Peninsula University of Technology, P.O. Box 1906, Cape Town, South Africa; ^4^Nursing Services Department, University of Benin Teaching Hospital, P.M.B. 1111, Benin City, Nigeria; ^5^Department of Biotechnology, Utkal University, Vani Vihar, Bhubaneswar, Odisha 751004, India; ^6^Department of Chemical Engineering, Faculty of Industrial Technology, Universitas Pembangunan Nasional “Veteran”, Yogyakarta, Indonesia; ^7^Department of Chemistry, Faculty of Science and Technology, Airlangga University, Mulyorejo, Surabaya 60115, Indonesia; ^8^Department of Mechanical Engineering, Faculty of Engineering, Edo State University Uzairue, P.M.B. 04, Auchi, 312101 Edo State, Nigeria

## Abstract

Presently, with the introduction of nanotechnology, the evolutions and applications of biosensors and/or nanobiosensors are becoming prevalent in various scientific domains such as environmental and agricultural sciences as well as biomedical, clinical, and healthcare sciences. Trends in these aspects have led to the discovery of various biosensors/nanobiosensors with their tremendous benefits to mankind. The characteristics of the various biosensors/nanobiosensors are primarily based on the nature of nanomaterials/nanoparticles employed in the sensing mechanisms. In the last few years, the identification, as well as the detection of biological markers linked with any form of diseases (communicable or noncommunicable), has been accomplished by several sensing procedures using nanotechnology vis-à-vis biosensors/nanobiosensors. Hence, this study employs a systematic approach in reviewing some contemporary developed exceedingly sensitive nanobiosensors alongside their biomedical, clinical, or/and healthcare applications as well as their potentialities, specifically for the detection of some deadly diseases drawn from some of the recent publications. Ways forward in the form of future trends that will advance creative innovations of the potentialities of nanobiosensors for biomedical, clinical, or/and healthcare applications particularly for disease diagnosis are also highlighted.

## 1. Introduction

The detecting of any disease (known as diagnosis in the medical terms) be it communicable (which result in about 4 million deaths annually around the world) or noncommunicable (causing over 70.00% of all deaths globally) is one of the dominant aspects toward the improvement of the efficiency of any biomedical/clinical/healthcare process [1–4]. According to the World Health Organization (WHO), the basic human diseases and infections are identified with the deficiency in access to good food and clean drinking water; explicitly, the utilization of hazardous water mostly from industrial activities represents about 80.00% of most diseases [5–7]. Hence, there is a need to continually search for diagnostic remedies to these diseases. Reportedly, the two basic prognoses in biomedical/clinical/healthcare diagnosis are the confirmation of the disease and the investigation of the vulnerability of an individual's as an age-linked category for several diseases. As rightly reported by the WHO, approximately 17.00% of every six deaths resulting from cancerous complications as well as other deadly diseases are caused by the late-phase detection and remote biomedical/clinical/healthcare diagnosis [8].

The treatment and convalescence for any kind of disease primarily depend on its early-phase detention as well as the effectiveness of the diagnosis processes [8, 9]. Microscopic procedures, immunosorbent approaches, and immunofluorescence (FRS) though demonstrated to be clinically critical in dealing with various diseases. However, they tend to exhibit certain limitations such as truncated sensitivity, stumpy-specificity, inaccuracy, expensiveness, and their cumbersome nature [10]. To mitigate these shortcomings, rapid, biocompatible, effective, and excellent throughput analytical procedures are now the evolving biomedical/clinical/healthcare necessities.

**Figure 1:**
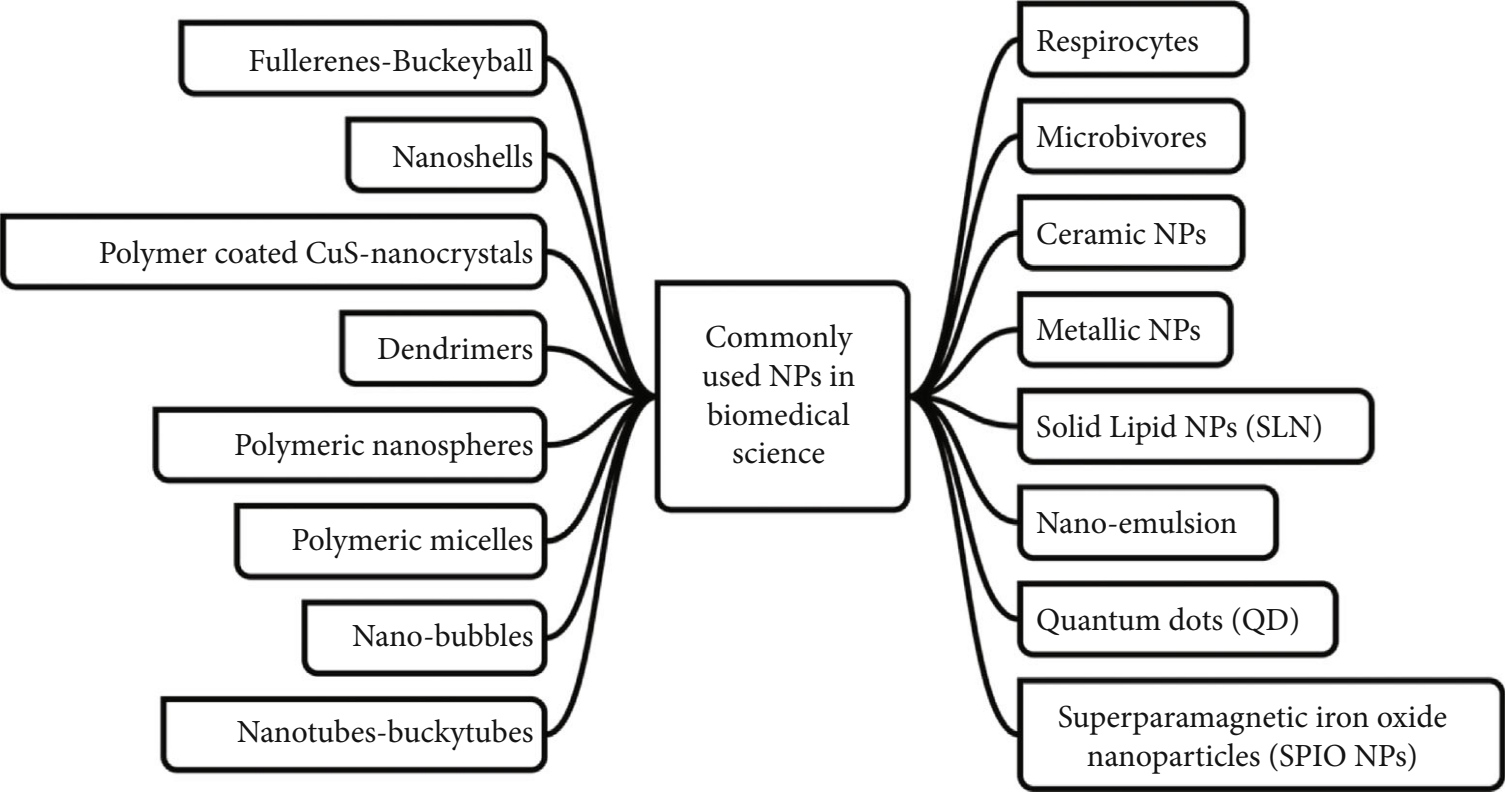
Diagrammatic illustration showing some of the commonest categories of NPs used for biomedical purposes [12].

Historically, the development of nanoparticles (NPs) commenced with the work of Ehrlich before the initial trials by Scheffel et al. as well as the all-embracing research work by some group of senior researchers led by Prof. Speiser at the ETH Zurich toward the end of the 1960s and beginning of the 1970s, with significant devotion to its development in the 1980s, particularly for medical purposes such as the delivery of drug voyage the blood-brain barrier (3Bs) [11, 12]. Presently, there are several reported categories of NPs, of which their magnitude (that is size and shape), as well as their origin to a large extent, is instrumental to determining their applications. Some of the commonest categories of NPs are shown in Figure 1 with particular reference to the ones used for biomedical purposes as represented in literature [12].

Supposedly, NPs are utilized as a means for delivering loaded constituents via two basic mechanisms: encapsulation (employing lipid-based/polymer-based capsules) and polymer-based components (employing natural/synthetic polymers) [12]. Generally, NPs have numerous benefits as compared to the conventional measures in the diagnostics/therapeutics fields. According to Jurj et al. [13], they are habitually harmless and biocompatible and can cross the 3Bs as well as other physiological constricts that serve as barriers [14]. Also, they could efficiently destroy intracellular and multiple drug-resistant pathogens [15], and they offer new procedures for the development of vaccines and gene treatments/rehabilitation (therapy) [16, 17].

Presently, biosensors (BioSS) are very significant for sensing target particles with great precision, selectivity, and signal-to-noise proportion. BioSS which are technologically advanced using BMs such as enzymes or nucleic acids (DNA/RNA) which are employed as the probes for sensing the target particles are presently been given great attention by several researchers owing to their numerous dynamic advantages. Enzymes that react with definite particles rapidly and selectively as well as the DNA/RNA can combine with their corresponding categorizations precisely in nanoscale [18]. Also, biomolecules (BMs) could immobilize and conjugate with other particles by modifying the surface via the relocation or introduction of chemical linkers [19, 20].

**Figure 2:**
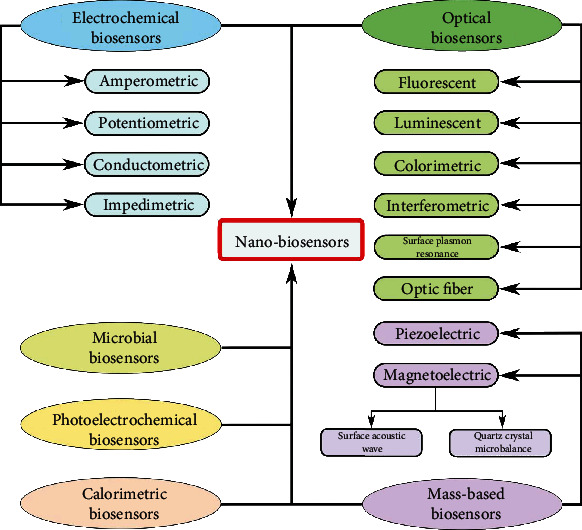
Some of the commonest categories and subcategories of BioSS [8].

**Figure 3:**
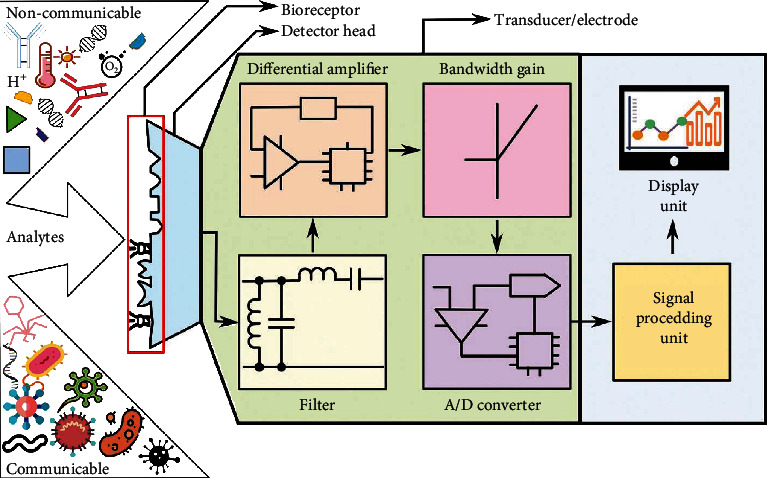
A block pattern of a practical BioSS for the detention of diseases [8].

**Figure 4:**
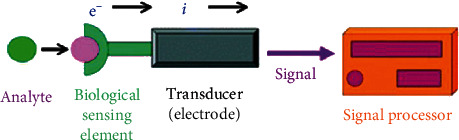
A distinctive diagram of an ETC BioSS [9].

Recently, the identification, as well as the detection of biomarker (BioMK) linked with any form of diseases, has been attained by several sensing procedures using nanotechnology (NanoTech) [21]. Some of these procedures comprise of electrodes with high conductivity that could identify or trace electron (particle) dynamic BMs or NPs, which are present in the body specifically for disease situations and for the generation of resilient signals [19]; all these stated characteristics are notable in sensing mechanism known as BioSS. These BioSS are generally characterized based on either the biological constituents (elements)/the category of the receptor that includes enzymes, cells and cell organelles, antibody (AB), or/and affinity receptors (DNA/RNA probes) and artificial (nonnatural) receptors or on the transducing constituents such as acoustic, calorimetric, electrochemical (ETC), and optical tendencies. BioSS can be characterized by the utilized transduction sensing procedure during its fabrication. The commonest categories and subcategories of BioSS are shown in Figure 2 [8]. However, Figure 3 shows a block pattern of a practical BioSS designed for the detention of diseases, and Figure 4 shows a distinctive diagram of an ETC BioSS.

Consequently, the implementation of BioSS is a beneficial approach for the protuberant detection of biological markers. Furthermore, the recent advancements in BioSS mechanism vis-à-vis nanobiosensor (NanoBioSS) have resulted in evolutionary modifications in various research fields, namely, agricultural, environmental, and biomedical/clinical/healthcare sciences as well as several other domains of human endeavours [22–28].

**Figure 5:**
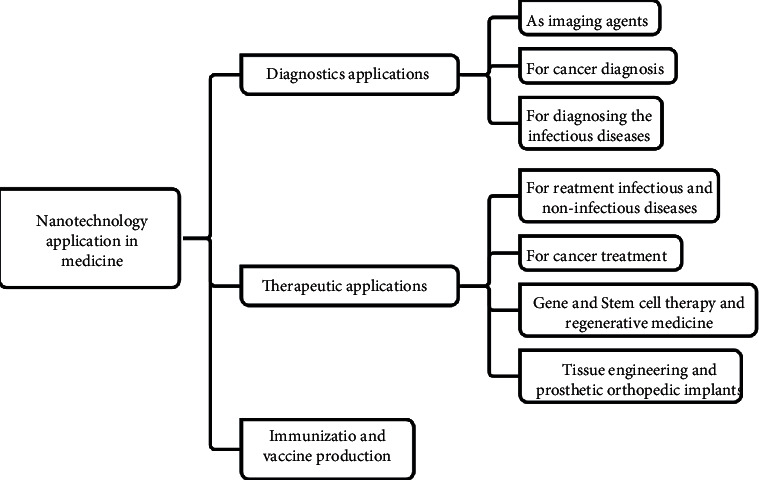
Some of the utmost applications of NPs for biomedical purposes [12].

Most of the preeminent applications of BioSS/NanoBioSS are found in the diverse manufacturing segments of which the biomedical/clinical/healthcare services are the primary ones [29, 30].  Figure 5 explores some of the utmost distinctive applications and proficiencies of NanoTech vis-à-vis BioSS/NanoBioSS that fall within the canopy of the biomedical/clinical/healthcare services as well as the allied services; the figure is a summary of NanoTech utilized for some of the utmost biomedical purposes such as diagnostic, therapeutics, and immunization [12].

The detection of diseases, retinal prostheses, cellular miRNA appearance in colorectal cancerous complications, imaging of contrast during MRIs, diagnosis of the heart, medicinal mycology, and the monitoring of health are the main momentous physiognomies or largely characterized areas well served with BioSS/NanoBioSS benefits [31–33]. These all-encompassing applications and proficiencies additionally improve the biomedical/clinical/healthcare services to an innovative pinnacle together with exceptional societal services [34–36].

Diverse irresistible diseases and infections spread such as Ebola, SARS, Hendra, Nipah, Avian influenza, and COVID-19 (SARS-CoV-2) have turned into a global threat that needs extensive exertion in their proliferation to manage. As there are diverse complications related to these irresistible diseases' infections, more advance diagnostic mechanisms need to be developed for mitigation and/or eliminating the odds of infection outbreak beforehand. BioSS/NanoBioSS has stood out as one of the appealing mechanisms for giving influential statistics on these diseases and infections. The recent SARS-CoV-2 plague (pandemic), which is extremely infectious, originated from a recently known coronavirus that has adversely obstructed humanity [37]. There have been some reported research studies on the application of BioSS/NanoBioSS in mitigating this dreaded virus [35, 38–41]. In the same way, innumerable other communicable and noncommunicable diseases such as Avian influenza, Ebola, Hendra, Nipah, and SARS have spawned substantial interest in recent times. Consequently, BioSS/NanoBioSS have enormous potential and proficiencies in detecting the outbreak of deadly virus together with any other diseases. Another great proficiency of the BioSS/NanoBioSS is in the diagnosis of the heart. Cardiovascular diseases are known as one of the utmost sources of death around the world, resulting in the death of over 17 million annually [2]. BioSS/NanoBioSS using BioMK is playing a critical role in the insurgency of diagnostic cardiovascular illnesses. The design and evolution of exceedingly sensitive and specific BioSS/NanoBioSS utilizing appropriate surface interactions and nanomaterials (NMs) are crucial for the specific diagnosis of heart illnesses [31–33, 42, 43].

**Figure 6:**
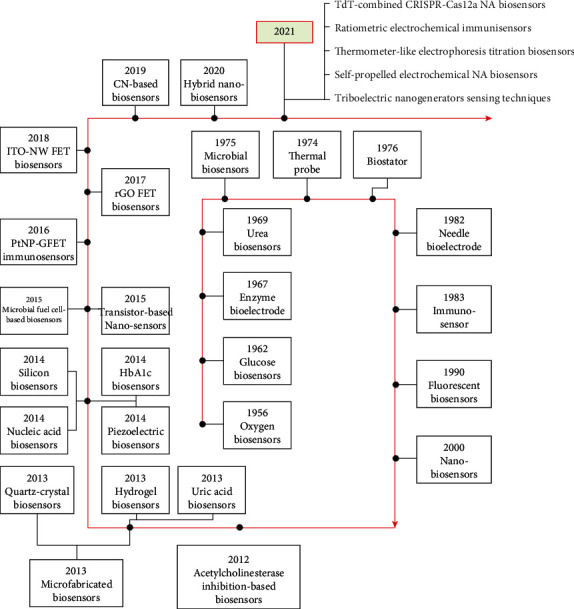
Historical background and advancements of some of the developments in BioSS/NanoBioSS [8] (FET: field effect transistor; GP: graphene; PtNP: platinum nanoparticle; rGPO: reduced graphene oxide; ITO-NW: indiumtin oxide nanowires; CN=: carbon nanotube; TdT: terminal deoxynucleotidyl transferase.

Over the years, several categories and subcategories of BioSS/NanoBioSS have been developed with vast applications (see Figure 2 for some of the main categories and subcategories of BioSS/NanoBioSS). Notwithstanding the effectiveness of most BioSS, there are still some reported limitations such as meagre selectivity, the influence of the charged constituent parts (particles) mostly in the form of interference, deficiency in the surface designs, and vulnerability to some environmental (ecological) interference [19, 44–46]. But with the evolutions of NanoTech (whose main concept deals with the execution of BMs or NPs, with an operational dimension of below 100 nm, in handling materials at the microscopic level [47]), some of these reported limitations are now been effectively moderated. According to [19], some of these critical limitations of BioSS are a result of variability and truncated signal strength resulting from the detector BMs. Henceforward, functional NMs assist in the mitigation of these limitations of BMs through the hybridization with or substitution of the BMs. Consequently, these functional NMs are beneficial for developing and evolving of the BioSS/NanoBioSS together with the increase of ETC signals, preservation of the actions of BMs for a lengthy duration, and advancement of investigating devices by the utilization of its distinctive plasmonic and optical possessions. Hitherto, numerous NMs have been produced and reported, ranging from broadly used Au NPs to innovative NMs that are either carbon-grounded or transition-metal dichalcogenide-grounded. These NMs were exploited either by themselves or through the hybridization (mixture) with other NMs for the development of highly sensitive BioSS/NanoBioSS [19].  Figure 6 shows some of the notable historical background and advancements of some of the reported developments for BioSS/NanoBioSS mechanisms as adapted from [8].

Reportedly, the performance of any BioSS/NanoBioSS is exceptional owing to their remarkable linearity, selectivity, sensitivity, and stability tendencies coupled with their outstanding response time and reproducibility as against the traditional BioSS. This evolving method is censoriously beneficial in the biomedical/clinical/healthcare domain as well as in clinical diagnosis. Hence, this study employs a systematic approach in reviewing some contemporary developed NanoBioSS together with their biomedical/clinical/healthcare applications and potentialities, specifically for the detection of some deadly diseases drawn from some of the recent publications. The study concludes by suggesting the way forward in the form of future trends that will advance creative innovations of the potentialities of nanobiosensors for biomedical, clinical, or/and healthcare applications particularly for disease diagnosis.

## 2. NanoTech in BioSS/NanoBioSS Mechanisms

BioSS/NanoBioSS are analytical devices that possess a biological sensor in addition to a physicochemical converter [22, 23, 28]. One of the major functions of any BioSS/NanoBioSS is to provide an incessant digital electrical signal that is comparative proportional to the summation of one or more ingredients that are being analysed [22].

BioSS/NanoBioSS are aiding some of the key advances in the analytics domains that are both assisting and being assisted by advances in NanoTech, implying that they represent both facilitating machinery and evolving applications in diverse fields. The capability of these BioSS/NanoBioSS to swiftly and precisely detect a substantial amount of NMs makes them vastly pertinent to a range of industrial, agricultural, ecological, and biomedical/clinical/healthcare as well as other scientific applications. Procedures to BioSS/NanoBioSS design/fabrication are as diverse as their applications, of which each of these BioSS/NanoBioSS categories has advantages and restrictions in the form of limitations based on the anticipated application, as well as the parameters that are essential for their optimum performance [23]. Hence, to be specific, the choice of BioSS/NanoBioSS design/fabrication should ruminate factors, for example, the sensitivity, specificity, dynamic range, output mode, activation time, usage simplicity, and engineering simplicity.

At the moment, BioSS/NanoBioSS are used in several aspects of human endeavours such as diagnosing different diseases and monitoring and management of the quality food and environmental effluences [8, 25, 28, 48, 49]. The surface dimension ratio of most frequently used NMs in BioSS/NanoBioSS such as noble metal NPs, quantum dot (QD), carbon-based NMs, and other NMs is larger when compared to the bulk arrangement of the material and this makes their properties (chemical, electrical, and optical) different and better enhanced [50]. These enhanced properties of NMs offer quicker detection and advanced reproducibility in NanoBioSS. Hence, NMs provide enhanced efficiency BioSS/NanoBioSS by improving the properties (ETC, mechanical and magnetic, and optical) of BioSS/NanoBioSS [51]. The fact that BioSS are more sensitive and compact today is achieved by including NMs in these bioanalytical devices.

**Figure 7:**
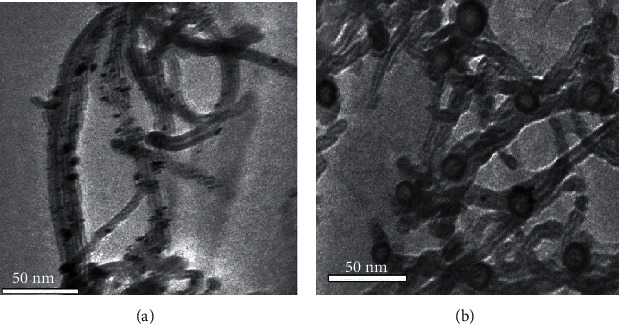
Images of TEM representing (a) a solid composite of Pt-supported multiwalled CN and (b) hollow composite of Pt-supported multiwalled CN [52].

For example, an innovative 3^rd^-generation glucose BioSS based on distinctive hollow PtNPs decorated with multiwalled CN (PtNPs-NT) composites was effectively fabricated. The PtNPs-NT composites were effectively arranged and directly cast on the glassy carbon electrode (GCE) surface. With the aid of electrostatic adsorption and covalent bonding, the negative (-) l-cysteine (l-cys) and the positive (+) poly (diallyl dimethylammonium) chloride- (PDDA-) coated gold (Au) NPs were improved on the ensuing surface of the electrode, which brought additional immobilization of glucose oxidase. Manipulation of the distinctive possessions of PtNPs-NT composites resulting in the accomplishment of direct transfer of electron among the electrode and the redox-active centres of glucose oxidase and the electrode demonstrated a couple of distinct reversible redox peaks with a fast heterogeneous rate of transfer of electrons [52] The images of the TEM representing a solid composite of Pt-supported multiwalled CN and the hollow composite of Pt-supported multiwalled CN are shown in Figure 7 [52].

**Figure 8:**
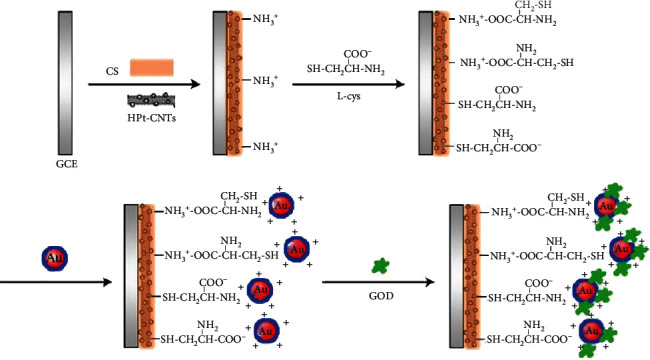
An illustrative representation of BioSS (Pt-CN) fabricated from multiwalled CN and hollow PtNPs-NT [52].

It is proven that CN has the prospective properties to transform several uses and benefits where nanosized metallic and/or semiconducting mechanisms are necessary [53]. For example, glucose BioSS combined with CN has been decorated with Au-coated Pd nanotubes [54], Au NPs [55], and Pt NMs [56]. Predominantly, Pt NMs with hollow interiors are auspicious due to their proficiencies to boost electron conveyance and upsurge the surface area. Spreading the surface area of the cathode powder is an active procedure for raising the activity of an electrode [57]. A BioSS on which the multiwalled CN coated with distinctive hollow nanostructure (NS) Pt has led to the accomplishment of direct transfer of electrons (Figure 8) [9].

**Figure 9:**
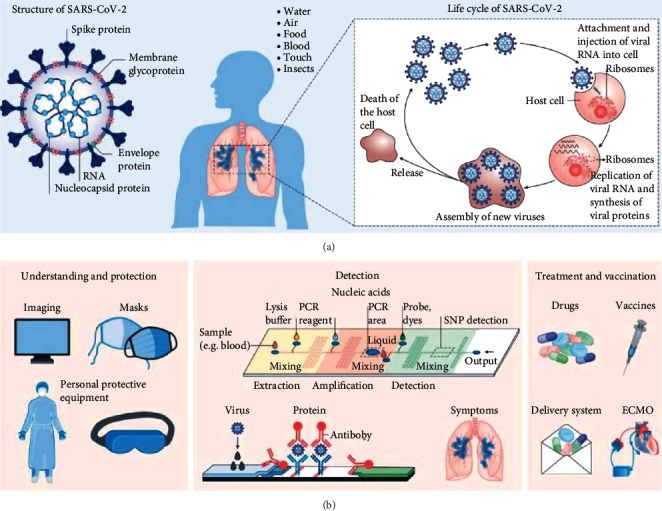
An illustration of the various roles of modern materials science employed during COVID-19 [65].

Several papers have reported on the uses of NanoTech vis-à-vis BioSS/NanoBioSS for biomedical/clinical/healthcare applications (such as identifying of viruses and pathogen microbes, detecting of cancerous cells, and breath analysis mechanism) [27, 58], environmental applications (detection of air, soil, and water pollution) [59–61], and agricultural applications (climate-smart organic agriculture and identification of animals and plants pests and diseases) [25, 48, 62–64]. There have also been suggestions on modern materials science vis-à-vis NanoTech been employed in COVID-19-related researches, as this has evidently played a dynamic role in mitigating and combating the present deadly COVID-19 complications via environmental remediation [37]. For example, Figure 9, as adapted from [65], illustrates the basic components, the various routes of transmission, and the duplication cycles of COVID-19 together with the utility of modern materials science in mitigating and combating the COVID-19 pandemic complications.

However, the major concentration of this facile review study is on the biomedical/clinical/healthcare applications of NanoTech vis-à-vis BioSS/NanoBioSS particularly for the detection of some deadly diseases drawn from some of the recent publications and this is being done in the subsequent section.

## 3. BioSS/NanoBioSS for the Detention and Treatment of Diseases

Even with the advancements in scientific knowledge, humanity is still fronted with some challenges ensuing from both communicable and noncommunicable diseases. As stated in Introduction, the prevention and early-phase detention as well as the effectiveness of the diagnosis and treatment processes are the most appropriate means for the survival and spread of such diseases. Hence, several innovative approaches such as the use of BioSS/NanoBioSS for the detention and treatment of diseases have continued to assist in this regard [8, 9, 51, 66–68].

According to several reports, presently, NanoTech innovations are felt in almost every scientific domain (such as biology, chemistry, computer science, environmental science, materials science, mathematics, physics, and engineering) and all the ensuing benefits (BioSS/NanoBioSS) are making life easier [47, 69–73]. Remarkably, in the last few years, NanoTech has been utilized in the monitoring and management of human health with auspicious results, specifically in the aspect of the treatment of cancerous complications [47, 74].

Table 1 and Table 2 encompass a summary of some studies involving the applications of BioSS/NanoBioSS for the detection of some of the most incapacitating diseases (Table 1 encompasses noncommunicable diseases, while Table 2 encompasses communicable diseases) drawn from some recent publications as adopted and modified from the broad and recent review publication work of [8].

However, the limit of detection (LoD) on this reported NanoBioSS varies and depends on some factors such as the utilized BioMK, the nature of the disease, the procedure employed in the BioSS mechanism, and the used BMs or NPs. Consequently, BioSS/NanoBioSS are predominantly characterized based on the nature of the NMs employed in the sensing mechanisms [19, 23, 26]. At the moment, there are, however, few reported commercialized BioSS for biomedical/clinical/healthcare applications; some of these are contained in Table 3 as adopted and modified from the broad review work of [8].

It was observed from Tables 1, 2, and 3 that the development of BioSS/NanoBioSS for medical purposes vis-à-vis disease detention is a contemporary dynamic aspect of modern material science (NanoTech). According to a recent review study by [26], the advancements of BioSS/NanoBioSS are fast attaining remarkable attention in the biomedical/clinical/healthcare fields due to their wide-ranging applications. BioSS/NanoBioSS are presently been efficaciously employed for detecting and diagnosing, treatment of diseases, as well as in the monitoring and management of human health [26, 95, 154–158]. Hence, there should be incessant advances in the development of materials (NMs to be specific) for the fabrication of BioSS/NanoBioSS.

## 4. Conclusion and Prospects of BioSS/NanoBioSS for the Detention of Diseases

In the recent past, the evolution of BioSS/NanoBioSS has remained as one of the dynamic areas of modern material science research (NanoTech) as attested by the large numbers of research publications. In the meantime, BioSS/NanoBioSS for detecting of diseases has stimulated a great deal of attention. The recent biomedical/clinical/healthcare applications (such as diagnostic, therapeutics, and immunization) of BioSS/NanoBioSS mechanism via the development of NanoTech present an encouraging procedure for the effective and precise detection of protein BioMK allied with various diseases. Be that as it may, this recent review study presents a facile review of some of the reported biomedical/clinical/healthcare applications and potentialities of NanoBioSS particularly for some deadly diseases, emphasizing some of the potential BioMK that could detect such diseases. As observed from most reported research publications, there are limited approaches that are concentrating in the direction for decreasing sample volumes or the duration of the analysis.

Consequently, there is still a great deal of work that needs to be carried out before NanoBioSS will be broadly employed in biomedical/clinical/healthcare laboratories as a replacement for just research laboratory purposes alone. A vibrant direction of imminent research is still in the aspect of molecular diagnostics for the accomplishment of advanced permanence and sensitivity. In the interim, diagnostic validation by processing an advanced quantity of biomedical/clinical/healthcare samples coming from persons infected with various diseases is required. Also, some components such as the nature of the protein, enzyme antigen, or/and other BMs, in addition to the concerned immobilization, should be considered. It is also suggested that the commercial approach to NanoBioSS from these useful reported researches should be one of the strategic aspects that require appropriate attention especially with funding and manpower in imminent research. Nevertheless, to exclusively achieve the biomedical/clinical/healthcare potentialities of NanoBioSS, additional and more researches should be executed and NanoBioSS could be pertinent in a complex matrix and extreme settings. Future research approaches should also hypothesize and conceptualize the implementations of innovative computational procedures such as big data analytics, Internet of Things, artificial intelligence, deep learning approachability, and microchip-built devices (all these are embedded in what is known as smart systems) interconnected with NanoBioSS for various biomedical/clinical/healthcare applications vis-à-vis the detection of diseases. Consequently, biomedical/clinical/healthcare investigations that recognise these smart systems interconnected with NanoBioSS should be reinvigorated for the development of prominent future detection of diseases (diagnostics).

## Figures and Tables

**Table 1:** Summary of some reported studies on BioSS/NanoBSS for the detection of some noncommunicable diseases.

BioMK	Diseases	Procedure	NPs used	LoD	Reference
Cancerous complications					
miRNA-182	Cancer of the lung	ETC	Molybdenum disulfide (MoS_2_)/Ti3C_2_ nanohybrids and modified GCE	0.43 fM	[75]
miR-106a and let-7a	Gastric cancer	ETC	AuNP and cadmium selenide (CdSe) @ cadmium sulphide (CdS) QTD-contained magnetic NCs (NCs) polythiophene/reduced graphene (GP) oxide-modified GCE	0.02 fM for let-7a and 0.06 fM for miR-106a	[76]
CXCL5	Colorectal cancerous cells CXCL5	ETC impedance spectroscopy (EIS) and voltammetry (VTM)	Chemokine receptor 2 (CXCR2) attached to conducting polymer-AuNP film	0.078 ± 0.004 ng/mL	[77]
miR-199a-5p	Triple-negative breast cancer (TNBC)	ETC	GCE with GP oxide (GPO) and Au nanorod	4.50 fM	[78]
HER-2	Breast cancer	ETC	AuNP grafted functionalized GP and NS polyaniline (PANI)	2 cells mL^−1^	[79]
miR-155	Breast cancer	ETC	GPO and Au nanorod	0.60 fM	[80]
BRCA1	Breast cancer	Cyclic VTM	ssDNA probe (BRCA1)/PANHS (polycyclic aromatic nitrogen heterocycles)/multiwalled CN/GCE	3.00 × 10^−18^ mol L^−1^	[81]
MUC1	Human non-small-cell lung cancerous cells	Amperometric (APM)	MUC1 aptamer probe and benzoic acid (TTBA) on AuNPs	8 cells/mL	[82]
MAGE A2	Lung cancer	ETC	Graphite/CN-chitosan/Ag (silver)/AB	5.00 fg mL^−1^	[83]
CpG islands of adenomatous polyposis coli (APC)	Colorectal cancer	FRS	Ferrosoferric oxide (Fe_3_O_4_)/Au core/shell NPs	3.10 × 10^−16^ M	[84]

Metabolic diseases					
Uric acid (UA)	Neuropapillitis, neurodegenerative diseases, sclerosis, and aplastic anaemia	ETC	Au/cobalt (Co) bimetallic NPs decorated hollow nanoporous carbon framework (Au/Co@HNCF)	0.023 *μ*M	[85]
Glucose	Diabetes (DBT)	ETC	Copper (Cu)-nanoflower decorated AuNPs-GO nanofiber (NF)	0.018 *μ*M	[86]
Vaspin	Type-2 DBT	FRS	Upconverting NPs (UC NPs)	39.00 pg mL^−1^	[87]
Ascorbic acid (AA), dopamine (DA), uric acid (UA), and acetaminophen (AC)	Scurvy, neurodisorders	ETC	Cerium oxide (CeO_2_) NPs-decorated CN	3.10 nM for AA, 2.60 nM for DA, 2.40 nM for UA, and 4.40 nM for AC	[88]
Vitamin D3	Rickets and cardiovascular diseases (CD)	ETC	Cu NPs-nickel NPs at reduced fullerene-C60 on GCE	0.0025 *μ*M	[89]
Leptin	Nonalcoholic fatty liver (NAFLD)	ETC	Bovine serum albumin (BSA)/anti-leptin/glutaraldehyde (Glu)/cysteamine (Cys)/AuNPs/porous GP (PGP)-BP (black phosphorus)/GCE immunosensor (IMSS) was employed	0.036 pg/mL	[90]
Glucose	DBT	ETC	Carbon quantum dot (CQD)/Au NPs and glucose oxidase (GOx) enzymes	17.00 *μ*M	[91]
3-Hydroxybutyrate (3-HB)	Hyperketonemia and diabetic ketoacidosis (DKA)	APM	Immobilization of the enzymes 3-hydroxybutyrate dehydrogenase onto a screen-printed GCE modified with GPO and thionine (THI)	1.00 *μ*M	[92]
Glucose	DBT	APM	Glucose oxidase immobilized on GPO-Fe_3_O_4_	0.10 *μ*M	[93]
Creatinine	Protracted kidney infection, CD, and type 2 DBT	APM	Immobilization of NPs of creatininase, creatinase, and sarcosine oxidase onto GCE	0.01 *μ*M	[94]

Neurological diseases					
Survival motor neuron (SMN) protein	Spinal muscular atrophy	VTM	Carbon NF-modified screen-printed electrodes	0.75 pg/mL	[95]
miR-195	Parkinson's disease	ETC	Exfoliated GPO and AuNWs were employed to amend the surface of screen-printed GCE	2.90 fM	[96]
APOe_4_	Alzheimer disease (AD)	FRS and ETC	Curcumin-GP QD platform coated on the transparent indium-tin-oxide electrode	0.48 pg mL^−1^	[97]
Amyloid-*β*	AD	FRS	Sheet-like structures of GP QD	Dependent on the FRS intensity	[98]
miR-145	Multiple sclerosis	FRS	Ag nanoclusters and hairpin oligonucleotide probes, MB1 and MB2	0.10 nM	[99]
*α*-1 Antitrypsin	AD	VTM	CN and Ag NPs functionalized with alkaline phosphatase-labeled AB	0.01 pmol L^−1^	[100]
Acetylcholine	AD	VTM	Extremely permeable Au electrode functionalized with acetylcholinesterase (AChE)	10.00 *μ*mol L^−1^	[101]
Amyloid-*β*	AD	ETC	Screen-printed GCE	0.10 ng/mL	[102]

Neonatal diseases					
C-reactive protein (CRP)	Sepsis	ETC	Magnetic reduced GPO/Ni (nickel)/platinum (Pt) NP micromotor biofunctionalization on the outer layer (using carbon black (CB), reduced GPO, multiwalled CN, and anti-CRP)	0.80 *μ*g/mL	[103]
Thyroid-stimulating hormone (TSH)	Thyroid dysfunctioning	ETC	Screen-printed GCE, anti-TSH AB, and amino-coated Ag NPs	0.001 *μ*IU/mL	[104]
Bilirubin (BR)	Jaundice	VTM	Reduced GPO oxide-poly styrene sulfonate (PSS) coated upon GCE	2.00 *μ*M	[105]

**Table 2:** Summary of some reported studies on BioSS/NanoBioSS for the detection of some communicable diseases.

BioMK	Diseases	Procedure	NPs used	LoD	Reference
Viral diseases					
Antibodies against COVID-19	COVID-19	Multiplexed grating-coupled FRS plasmonics	Au-coated nanoscale	1 : 1600 dilution	[106]
Dengue viral RNA	Dengue virus	ETC monitoring	Methylene blue conjugated AuNPs	100.00 fM	[107]
S spike glycoproteins	SARS-CoV-2	ETC monitoring	GPO and Au nanostars	1.68 × 10^−22^ *μ*g mL^−1^	[108]
Peptide DNA/RNA	Influenza A viruses (H1 to H16 subtypes)	Visual colorimetric assay (CMA)	Au NPs	2.30 ng	[109]
DENV proteins	Dengue viral disease	ELISA-plate spectrophotometers	Au nanorods	1.00 pg	[110]
COVID-19 spike protein	COVID-19	FET-based BioSS	GP sheets	2.42 × 10^2^ copies/mL	[111]
Complementary sequences of RdRp-COVID-19, ORF1ab-COVID-19, and E genes of COVID-19	COVID-19	PPT effect and LSPR sensing transduction	Dual-dimensional Au nanoislands (AuNIs)	0.22 pM	[112]
HBV DNA	Hepatitis B	ETC impedance spectra (EIS)	Tin-doped WO3/In2O3 nanowires	0.10 pM to 10.00 *μ*M	[113]
Virus DNA/RNA	Narrowly related Zika and dengue viruses	Fluorometric detection	GPO	2.10 × 10^1^ − 5.1 × 10^2^ FFU/mL	[114]
Dengue viral DNA	Dengue viral disease	Sandwich hybridization strategy of DNAs	AuNPs	1.00 × 10^−29^ M	[111]
Sialyl oligosaccharide receptor-mimic peptide	Influenza A virus	Optimized peptide termination	Boron-doped diamond electrode	5.00–10.00 pfu/sample	[115]
HCVcoreAg	Hepatitis C	Modification of buffer pH from acidic to neutral	Silicon-on-insulator (SOI) nanowire	0.30 pg/mL	[116]
Concanavalin A lectin	Dengue type 2, Zika, chikungunya, and yellow fever	Cyclic VTM and impedance spectroscopy	Zinc oxide NPs	0.0421 pfu/mL for ZIKV, 0.0437 pfu/mL for YFV, 0.062 pfu/mL for CHIKV, and 0.0382 pfu/mL for DENV	[117]
l-lysine levels	HIV	APM BioSS	l-lysine oxidase (LOx NPs) and GPO NPs	0.01 *μ*M	[113]
Nonspecific proteins	MERS-CoV and HCoV	Electrochemiluminescence	Au NPs	0.40 and 1.00 pg mL^−1^ for HCoV and MERS-CoV, respectively	[118]
Hepatitis B virus gene	Hepatitis B	ETC monitoring	AMT-Au NPs-PGEs	0.86 *μ*g/mL	[119]
Viral DNA	HPV-18	FRS assay	Ti_3_C_2_ nanosheets	100.00 pM	[120]
HIV-1 gene	AIDS	Electrochemiluminescence NanoBioSS	Europium sulfide nanocrystals (EsNCs)	3.00 fM to 0.30 nM	[121]
Envelop protein AB (Zev-Abs)	Zika virus	ETC IMSS	Interdigitated microelectrode of Au (IDE-Au)	10.00 pM	[122]
Virus oligonucleotide	MERS-CoV	CMA	Citrate anion-stabilized AgNPs	1.53 nM	[123]
Virus oligonucleotide	Human papillomavirus	CMA	Citrate anion-stabilized Ag NPs	1.03 nM	
Surface receptor	Influenza A	Chromatographic assay	Carbon NPs	350 TCID50/mL (i.e., the 50% tissue culture infectious dose)	[124]
JEV via recognition cavities	Japanese encephalitis virus	FRS detection	Magnetic silicon microspheres	2.50–45.00 nM	[125]
	Influenza A (H_1_N_1_) and A (H_3_N_2_)	Paper-based immunoassay (IMA)	Au NPs	2.70 × 10^3^–2.70 × 10^4^ plaque-forming unit per assay	[126]
AB specific to influenza virus	Influenza A (H_7_N_9_)	ETC sensor	GPO, multiwalled CN	0.81 pg/mL	[127]
AB specific to viral infection	Influenza A and B	IMA	Europium NPs	1.00 × 10^1^ to 1.00 × 10^3^ EID 50/mL	[128]
Specific mouse *α*-A NP mAbs	Influenza A (H_1_N_1_)	FRS IMA	Magnetic NPs (MnFe_2_O_4_)	0.007 HAU	[129]
	Influenza A (H_3_N_2_)	FET BioSS	Silicon nanowire, magnetic NPs	29 viruses/*μ*L	[130]
DNA-based detection	Influenza A (H_5_N_1_)	DNA-based microarray assay (scanometric detection)	AuNPs with Ag staining technique	1.00 × 10^2^ fM per assay (PCR fragments) 1.00 × 10^3^ TCID50 per assay (viral RNA)	[131]

Bacterial diseases					
Bacterial target DNA	*S. aureus*	Targeted DNA was quantified in spectrophotometry at 260 nm; the sensitivity of this method was studied with PCR and gel agarose electrophoresis	MNP-TiO_2_-AP-SMCC	230.00 CFU/mL	[132]
Electrostatic interaction of cell wall and concomitant inhibition of peroxidase activity of CS-MNPs	Gram-negative *Escherichia coli* or the Gram-positive *Staphylococcus aureus*	CMA	Chitosan-coated iron oxide magnetic NPs (CS-M NPs)	1.00 × 10^4^ CFU/mL by the naked eye and 1.00 × 10^2^ CFU/mL by spectrophotometry within 10 min	[133]
Anti-*E. coli* O157 AB	*E. coli* O157	Cyclic VTM and ETC impedance spectroscopy	Au NPs	15.00 CFU/mL	[134]
Anti-*E. coli* AB	*E. coli*	Chemiresistive BioSS	Au NPs	12.00 CFU/mL	[135]
Biofilm	*Staphylococcus epidermidis*	ETC sensing	Magnesium zinc oxide (MZO) NS	A drain current change of ~80% after ~200 min of *S. epidermidis* bacteria culturing	[136]
Bacterial peptides	*Listeria monocytogenes* and *Staphylococcus aureus*	ETC BioSS	Au NPs	3.00 CFU/mL for *Staphylococcus aureus* and 9.00 CFU/mL for *Listeria monocytogenes*	[137]
Bacteria's target DNA	Foodborne bacteria including *Escherichia coli* O_157_:H_7_, Vibrio parahaemolyticus, Salmonella, Staphylococcus aureus, Listeria monocytogenes, Shigella, etc.	Amplified microcantilever array BioSS	Au NPs	0.005–0.040 fM or 1–9 cells/mL	[138]
Receptor-binding protein of bacteria	*Escherichia coli*, *Pseudomonas aeruginosa*, and *Vibrio cholerae*	CMA	Au NPs	∼100 cells	[139]
Mycobacterium tuberculosis oligonucleotide	Mycobacterium tuberculosis (MTB)	CMA	Citrate anion-stabilized (Ag NPs)	1.27 nM	[123]
Fungal diseases					
Fungal spores	*Aspergillus niger*	CMA	Peptide-modified Au NPs	50 spores	[140]
Concanavalin A (ConA) and wheat germ agglutinin (WGA) lectins	*Candida spp.*	Impedimetric BioSS	Lectin-modified Au NPs	1.00 × 10^2^–1.00 × 10^6^ CFU/mL	[141]
Protein BioMK	*Aspergillus fumigatus* allergen Asp f 1	CMA	Magneto-BioSS biochip	~100.00 pg/mL	[142]

Parasitic diseases					
AB as receptor	Malaria	ETC BioSS	Platinum NPs (Pt NPs)	8.00 ng/mL	[143]
pLDH	Malaria	EIS: ETC impedance spectroscopy	GCE	0.50 fM	[144]
*β*-Hematin	*P. berghei*, *P. falciparum*	ETC NS	Au-CuO	3.60–4.80 mM 0.65–1.35 mM	[145]
Bilharzia AB	Bilharzia disease	ETC NanoBioSS	Nanostrip with immobilized Au NPs	8.39 × 10^–2^ ng/mL	[146]

**Table 3:** Some reported commercialized BioSS/NanoBioSS for biomedical/clinical/healthcare applications.

Target analyte	Linked disorder	Type of BioSS/NanoBioSS	Reference
Glucose	DBT	Enzymatic-ETC NanoBioSS, lateral flow (LF) immunochromatographic (ICM) assays reverse iontophoresis	[147]
Human chorionic gonadotropin (hCG)	Gestation, fertility, and ovulation	LF ICM assay, FRS-labeled AB assay	[148]
*Streptococci spp.*	Diseases of the throat or skin	LF ICM assay, FRS-labeled AB assay	[149, 150]
*Mycobacterium tuberculosis*	Tuberculosis	LF ICM assay, FRS-labeled AB assay	[151]
Alpha-fetoprotein (AFP)	Cancerous complications	LF ICM assay, ETC	[152]
*Bacillus anthracis*	Anthrax	Standard LF assay, FRS-labeled AB assay	[153]

## Data Availability

Completely, data produced or investigated during this work were involved in this submitted article.

## Abbreviations

APM: Amperometric
AB: Antibody
BioMK: Biomarker
BMs: Biomolecules
BioSS: Biosensor
3Bs: Blood-brain barrier
CN: Carbon nanotube
CMA: Colorimetric assay
ETC: Electrochemical
FET: Field-effect transistor
FRS: Fluorescence
GCE: Glassy carbon electrode
GP: Graphene
ICM: Immunochromatographic
IMSS: Immunosensor
IMA: Immunoassay
ITO-NW: Indiumtin oxide nanowires
LF: Lateral flow
LoD: Limit of detention
NanoBioSS: Nanobiosensor
NCs: Nanocomposites
NF: Nanofiber
NMs: Nanomaterials
NPs: Nanoparticles
NanoTech: Nanotechnology
NS: Nanostructure
PtNP: Platinum nanoparticle
rGPO: Reduced graphene oxide
QD: Quantum dot
TdT: Terminal deoxynucleotidyl transferase
VTM: Voltammetry.
